# Synthesis of Quantum Dot-ZnS Nanosheet Inorganic Assembly with Low Thermal Fluorescent Quenching for LED Application

**DOI:** 10.3390/ma10111242

**Published:** 2017-10-27

**Authors:** Yangyang Xie, Chong Geng, Yiqun Gao, Jay Guoxu Liu, Zi-Hui Zhang, Yonghui Zhang, Shu Xu, Wengang Bi

**Affiliations:** 1 Tianjin Key Laboratory of Electronic Materials and Devices, School of Electronics and Information Engineering, Hebei University of Technology, 5340 Xiping Road, Beichen District, Tianjin 300401, China; xyyhebut@163.com (Y.X.); gengchong@hebut.edu.cn (C.G.); zh.zhang@hebut.edu.cn (Z.-H.Z.); zhangyh@hebut.edu.cn (Y.Z.); 2 SHINEON Co., LTD., Building 3, No. 58 Jinghai Road, BDA, Beijing 100176, China; 102996857@163.com (Y.G.); jayliu@shineon.cn (J.G.L.)

**Keywords:** quantum dots, nanosheet, assembly, thermal stability

## Abstract

In this report, to tackle the thermal fluorescent quenching issue of II-VI semiconductor quantum dots (QDs), which hinders their on-chip packaging application to light-emitting diodes (LEDs), a QD-ZnS nanosheet inorganic assembly monolith (QD-ZnS NIAM) is developed through chemisorption of QDs on the surface of two-dimensional (2D) ZnS nanosheets and subsequent assembly of the nanosheets into a compact inorganic monolith. The QD-ZnS NIAM could reduce the thermal fluorescent quenching of QDs effectively, possibly due to fewer thermally induced permanent trap states and decreased Förster resonance energy transfer (FRET) among QDs when compared with those in a reference QD composite thin film. We have demonstrated that the QD-ZnS NIAM enables QDs to be directly packaged on-chip in LEDs with over 90% of their initial luminance being retained at above 85 °C, showing advantage in LED application in comparison with conventional QD composite film.

## 1. Introduction

For the last decade, semiconductor quantum dots (QDs) have drawn constant research interest in many applications, such as light-emitting diodes (LEDs), bio-sensing, and solar cells [1,2,3]. Compared with traditional inorganic phosphors and organic dyes, QDs have unique optical properties, including broad absorption spectra, tunable and narrow emission profiles, high quantum efficiency, and high resistance towards photo bleaching [4,5,6]. Therefore, they have been considered promising alternatives for next-generation high-performance lighting and display devices, such as QDTV with QD-enhanced LED backlighting. Nevertheless, the practical application of QDs in LEDs has been seriously restricted because of the LED packaging issue. One major reason is that these nano-sized QDs are very sensitive to ambient environment and temperature, leading to considerable thermal fluorescent quenching and degradation [7,8]. Researchers have made continuous efforts to solve the problem through embedding QDs in polymer matrices, or growing inorganic barrier layers, such as silicon and alumina, to protect QDs from the ambient environment [9,10,11,12]. Nevertheless, all of the above methods have to employ complicated QD surface-treatment processes, and the thick barrier layers usually cause strong light scattering and a degree of degradation in optical performance, making them less economically suitable for LED applications.

As an important semiconductor, zinc sulfide (ZnS) has been utilized as a barrier layer for CdSe and CdS QDs to confine the carriers due to its wide band gap and relatively high photochemical stability. Nevertheless, it is rather difficult to grow a thick ZnS barrier layer around QDs because of the lattice mismatch; otherwise, the interface lattice stress will create a large number of defects and lead to degradation of quantum efficiency. Therefore, it is highly desired to have a new and simple method to protect QDs from thermal fluorescent quenching. Meanwhile, researchers have paid attention to other low-dimensional nanostructured materials, such as 1D nanotubes and 2D structural nanosheets, in order to involve their unique physical properties in applications in optics, magnetics and electronics [13,14,15,16]. Such low-dimensional materials often exhibit higher stability than QDs due to their relatively low surface-to-volume ratio. Similar to other 2D structural semiconductors, ZnS nanosheet possesses enhanced stability over QDs because their relatively low surface-to-volume ratio provides fewer defects and reduces surface reactivity [16]. Therefore, it may offer the opportunity to reduce thermal fluorescent quenching of QDs through growing a reversed structure via surface adsorption of QDs onto ZnS nanosheet and subsequent assembly of the QDs and ZnS nanosheet into a multi-layered 2D assembly.

For this purpose, we propose a QD-ZnS nanosheet inorganic assembly monolith (QD-ZnS NIAM) to improve the photoluminescence (PL) efficiency and reduce thermal fluorescent quenching for CdSe/CdS/ZnS core-shell QDs. The 2D ZnS nanosheets are synthesized via a low-temperature hydrothermal route and subsequently employed as templates for the chemisorption of the QDs onto their surfaces. In line with our expectations, we demonstrate that the layer-by-layer (LBL) assembly of the QDs and the 2D ZnS nanosheets into a compact inorganic monolith could effectively reduce the thermal fluorescent quenching for the QDs. Furthermore, the as-prepared monolith shows considerable stability enhancement at high temperature in comparison to commercial QDs. In addition, the QD-ZnS NIAM can be easily integrated into LEDs, and provides low light scattering, thus improving light extraction. The proposed procedure avoids complicated surface treatment processes for QDs, and has additional advantages in terms of light extraction and reproducibility.

## 2. Materials and Methods

### 2.1. Materials

Zinc acetate dehydrate (Zn(CH_3_COO)_2_·2H_2_O, 98%) was purchased from Alfa Aesar (China Chemicals Co., Ltd., Shanghai, China). N-octylamine (OCA, 99%) was purchased from Shanghai Macklin Biochemical Co., Ltd. (Shanghai, China). Sodium sulfide pentahydrate (Na_2_S·5H_2_O, AR) was purchased from Tianjin Damao Chemical Reagent Co., Ltd. (Tianjin, China). Ethylenediaminetetraacetic acid (EDTA, AR, 99.5%) was purchased from Aladdin Reagents (Aladdin Industrial Corporation, Shanghai, China). The QDs were purchased from Mesolight Inc. (Suzhou, China) with quantum yields of 85%. Other organic solvents were purchased from Sinopharm Chemical Reagent Co., Ltd. (Shanghai, China). All chemicals were used directly without any further purification unless otherwise stated.

### 2.2. Characterization

UV-Vis absorption was measured by using a Persee T6 UV-Vis spectrometer (Beijing Persee General Instrument Co., Ltd., Beijing, China). Photoluminescence (PL, Shanghai Ideaoptics Phenix Optical Company Limited, Shanghai, China) was recorded on an Ideaoptics FX2000-EX PL-spectrometer. Transmission electron spectroscopy (TEM) was performed on a FEI Tecnai G2 Spirit TWIN transmission electron microscope operating at 100 kV (FEI Company, Hillsboro, OR, USA). Scan electron microscopy (SEM) experiments were performed on FEI Nova NanoSEM 450 (FEI Company, Hillsboro, OR, USA). X-ray diffraction (XRD) experiments were realized with a SmartLab X-ray diffractometer (Rigaku Corporation, Tokyo, Japan). EDS (energy-dispersive spectrometer) characterization was performed on an EDAX Octane Plus Det operating at 1.76 keV (EDAX Company, Mahwah, NJ, USA).

### 2.3. Synthesis of Zinc Sulfide (ZnS) Nanosheets

ZnS nanosheets were synthesized by a low-temperature solution-precipitation method with some modifications [15]. 1.095 g of Zn(CH_3_COO)_2_·2H_2_O and 1.60 g of EDTA were mixed in 50 mL of distilled water, together with 5 mL of OCA to form a clear liquid. Then, 1.56 g of Na_2_S·5H_2_O were mixed in 50 mL of distilled water and the mixture was stirred for another 20 min to ensure the reaction was completed. Then, the mixture was added to the above liquid. After stirring for 5 min, the mixture was heated to 85 °C. The mixture was maintained at 85 °C for 3 h and air-cooled to room temperature. After the reaction, a white precipitate was formed. The precipitate was washed with distilled water, and then with absolute ethanol repeatedly to remove inorganic and organic residues. The final product was obtained by drying overnight under vacuum.

### 2.4. Synthesis of QD-ZnS NIAM

100 mg ZnS sheet powders were added into 1 mL QDs-toluene solution in a 10 mL centrifuge tube. And then, 5 mL absolute ethanol were added. After 20 min ultrasonic vibration, the mixture solution was centrifuged (9000–10,000 rpm) for 10 min. Then, the products in orange solid form were obtained by drying 1 h under vacuum. After being pulverized, the products were pressed into thin slice via a bead machine. Finally, the QD-ZnS NIAM was obtained.

## 3. Results and Discussion

**Figure 1:**
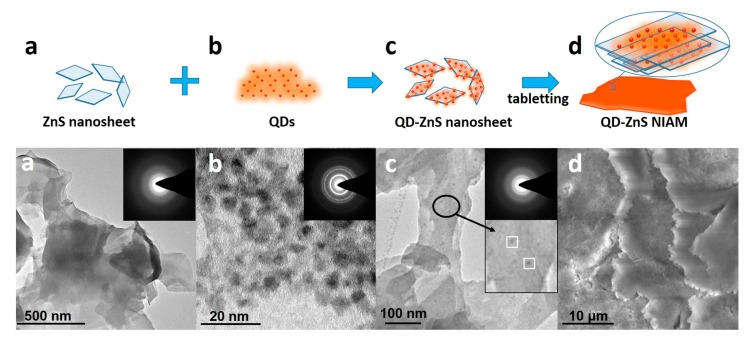
Illustration of the preparation procedure for the QD-ZnS nanosheet inorganic assembly monolith (QD-ZnS NIAM) and the TEM images of (**a**) the ZnS nanosheet; (**b**) QDs; (**c**) QD-ZnS nanosheet and (**d**) the SEM image of the QD-ZnS NIAM.

Figure 1 shows the formation procedure of the QD-ZnS NIAM. The sample at each stage is detected by transmission electron microscopy (TEM), as shown in Figure 1a–c, and by scanning electron microscope (SEM), as shown in Figure 1d. First, the ZnS nanosheets are synthesized via a low temperature hydrothermal route, as shown by the crosslinked large thin films in the corresponding TEM image in (a). Figure S1 shows the XRD characterization of the ZnS nanosheet. The diffraction peaks can be indexed to a wurtzite ZnS (JCPDS Card 36-1450). Next, the QD-ZnS nanosheet assembly is developed through mono-dispersed chemisorption of the commercial QDs (b) on the surface of the 2D ZnS nanosheets after stirring their mixture in solvent. The TEM image of QD-ZnS nanosheet (c) clearly exhibits adsorption of QDs on the nanosheet surface, as indicated by the QDs with a diameter of about 7 nm and ZnS nanosheet with side length more than 100 nm in the insert image in c. Moreover, there is almost no QD aggregation on the nanosheet surface for the same size distribution of QDs (shown in (c)). Finally, the QD-ZnS NIAM (d) is prepared via a tableting procedure. In detail: an appropriate amount of QD-ZnS nanosheet (c) powder is put into a mold, and the QD-ZnS NIAM (d) is formed after pressing the powder into a solid tablet under 2 times atmospheric pressure for 5 minutes. The SEM image clearly shows the morphology of the LBL assembly of the QD-ZnS nanosheet in the compressed monolith. The EDX measurement of the QD-ZnS NIAM shown in Figure S2 gives a Zn:S:Cd:Se atomic ratio of 46.21:50.21:2.02:1.56, which indicates the calculated amount of CdSe/CdS/ZnS QDs in the NIAM with approximate 13.5% in weight according to the size of the QDs.

**Figure 2:**
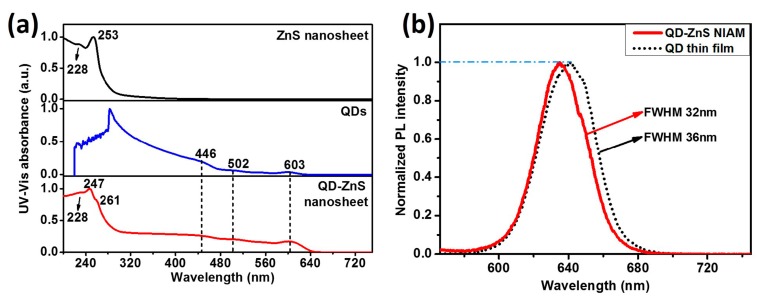
(**a**) Ultra-violet-visible (UV-Vis) absorption spectra of ZnS nanosheet (powder), QDs (solution) and QD-ZnS nanosheet; (**b**) Normalized photoluminescence (PL) emission spectra of QD-ZnS NIAM and QD thin film (excited by a 440 nm laser).

The optical properties of ZnS nanosheet and QDs-ZnS nanosheet are exhibited in the UV-vis absorption and PL emitting spectra, as shown in Figure 2. UV-vis absorption of the ZnS nanosheet powders shows two obvious absorption peaks at 253 nm and 228 nm, respectively. The large blue shift of the peaks in comparison with that of bulk ZnS (344 nm) gives the evidence of quantum confinement effect and indicates that the thickness of the as-prepared nano-structural ZnS sheet is much smaller than the exciton bohr radius of ZnS. Obviously, in Figure 2a, the QDs-ZnS nanosheet structure obtains the combined absorption peaks of QDs and ZnS nanosheet, indicating that there is no band correlation or interaction between the two materials. Figure 2b compares the PL spectra of the QD-ZnS NIAM and spin-coated QD thin film on glass. The assembly shows a narrower full width at half maximum (FWHM) and a relatively blueish emission peak, indicating good dispersion of QDs in the QD-ZnS NIAM.

**Figure 3:**
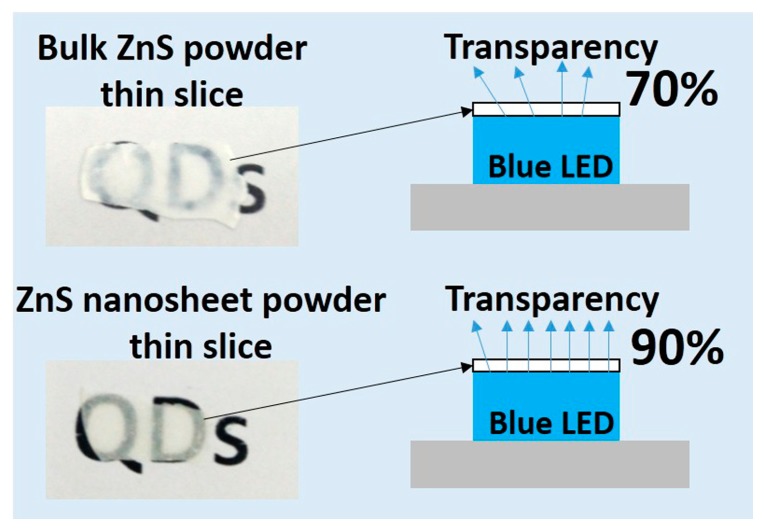
Light transparencies of ZnS nanosheet and bulk ZnS powder thin slices in LEDs.

**Figure 4:**
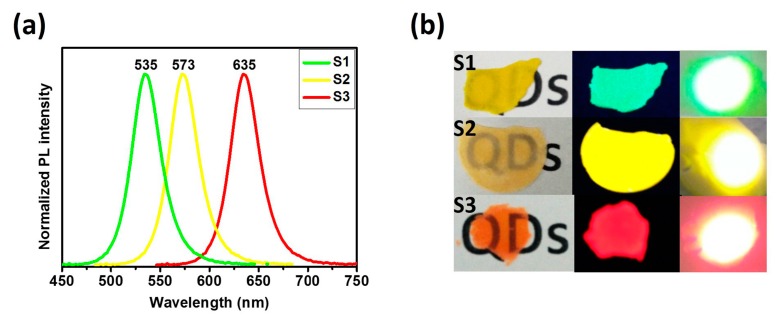
(**a**) Normalized PL emission spectra of the monochromatic QD-ZnS NIAM color converters; (**b**) Monochromatic QD-ZnS NIAM color converters under roomlight, UV light and 440 nm laser irradiation.

Moreover, the ultra-thin ZnS nanosheet structure provides high light transparency. Since the ZnS nanosheet in the LBL assembly is highly oriented, the NIAM could achieve low light scattering on the vertical direction, which would benefit the overall light extraction. Figure 3 compares the blue-light transparency of the monolith thin slices compressed from bulk ZnS powder and from the ZnS nanosheet, respectively. Both of the two samples have the thickness of 0.5 mm, which was measured by a vernier caliper. The ZnS nanosheet assembly monolith displays much higher light extraction (about 90%) than that of the compressed bulk ZnS powder (70%) with the same thickness and LED package. Hence, the QDs are embedded within the ZnS nanosheet to form a highly transparent and multi-layered sandwich structure in the QD-ZnS NIAM. The as-developed structure plays roles in both protecting the QDs inside the assembly and providing high light-extraction efficiency. A group of monochromatic QD-ZnS NIAM as color converters are shown in Figure 4b with their PL emission spectra shown in Figure 4a, which show high transmittance and color saturation. The transparent QD-ZnS NIAM thin slices under daylight as shown in Figure 4b give the evidence of the high photopermeability.

**Figure 5:**
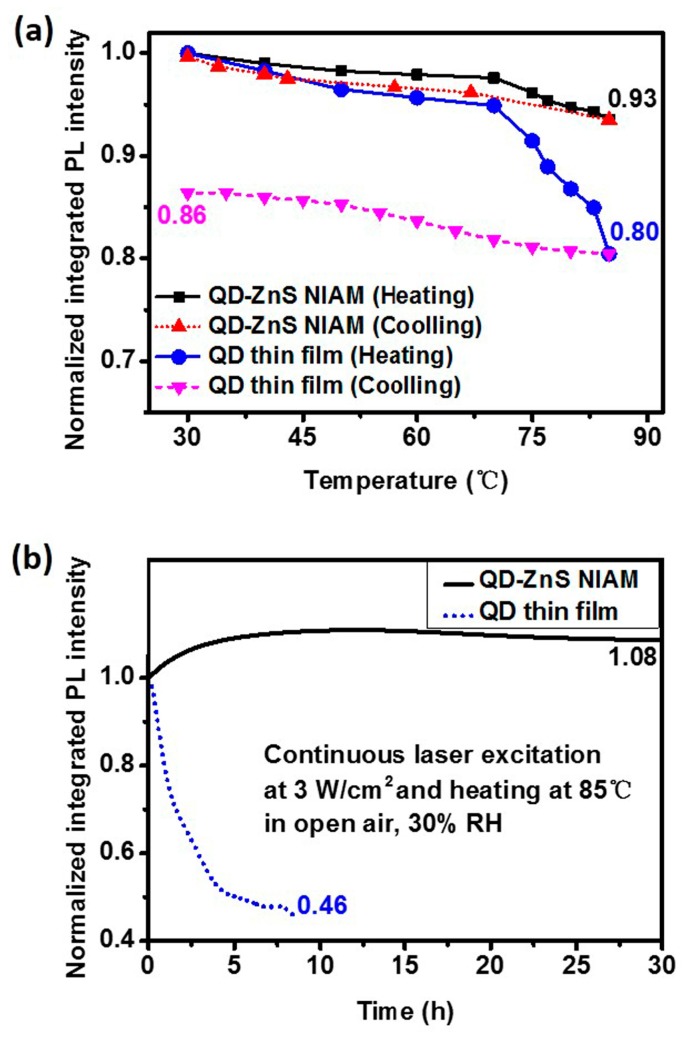
(**a**) The thermal quench and PL reversibility test results of the QD-ZnS NIAM and the QD thin film; (**b**) Photochemical stability test results of the QD-ZnS NIAM and the QD thin film at 85 °C and 30% relative humidity (RH) under continuous laser excitation in air.

The QD-ZnS NIAM structure has exhibited the capability that notably reduce thermal fluorescent quenching and improve the photochemical stability of QDs. Figure 5a shows the thermal quenching and PL reversibility test results for the red QD-ZnS NIAM and the QD thin film. The two samples contain the same amount of QDs. The QD-ZnS NIAM shows about 7% in thermal fluorescent quenching when the temperature rises from room temperature to 85 °C, and almost returns to its original PL intensity after cooling. In comparison, the QD thin film exhibits considerable quenching when the temperature raises over 65 °C, with a record of 20% thermal fluorescent quenching at 85 °C, and much worse PL reversibility, with only 86% of its original PL value being restored. Figure 5b shows the change of PL intensity of the QD-ZnS NIAM and the QD thin film at 85 °C and 30% RH under continuous laser excitation, which could indicate the photochemical stability of the two materials. The QD-ZnS NIAM becomes photochemically stable for a stable PL intensity after an initial PL enhancement stage for approximate 5 h. As a reference, the photoluminescence of the QD thin film starts degradation at about 14% per hour, and decays by 54% within 9 h.

This result demonstrates that the QD-ZnS NIAM can effectively improve the thermal and photochemical stability of QDs. We consider the improvement to be attributable to the following three reasons. First, according to a previous study, it could be explained by there being fewer thermally created permanent trap states in the QD-ZnS NIAM [17]. Since the crystalline ZnS has a thermal conductivity coefficient of 25 w/(m·k), the ZnS nanosheet has a much higher thermal conductivity along the 2D direction than organic ligands on the QD surface and polymer in the LED package. It provides rapid heat dissipation from QDs to the nearby environment to effectively reduce the heat accumulation for QDs under continuous irradiation. For this reason, thermally created trap states can be greatly reduced [18]. Moreover, QDs are mono-dispersed, and are fixed on the surface of ZnS nanosheet via chemisorption with an average distance of over 10 nm, as observed in the TEM image. The distance inhibits the Förster resonance energy transfer (FRET) within QDs, thus reducing the nonradiative recombination caused by the FRET [19,20]. Furthermore, the elevated pressure in the QD-ZnS NIAM helps to transfer the assembly from a metastable structure to a stable structure that increases the overall stability, according to the literature [21]. In addition, the PL enhancement at the beginning can be explained by the reduced nonradiative recombination rates in QDs through thermal annealing according to reported studies [22,23]. Nevertheless, further investigation into the interface of the materials in NIAM will be necessary in order to understand the thermal quenching mechanism and the role of the ZnS nanosheet in the assembly from the standpoint of physicochemistry.

**Figure 6:**
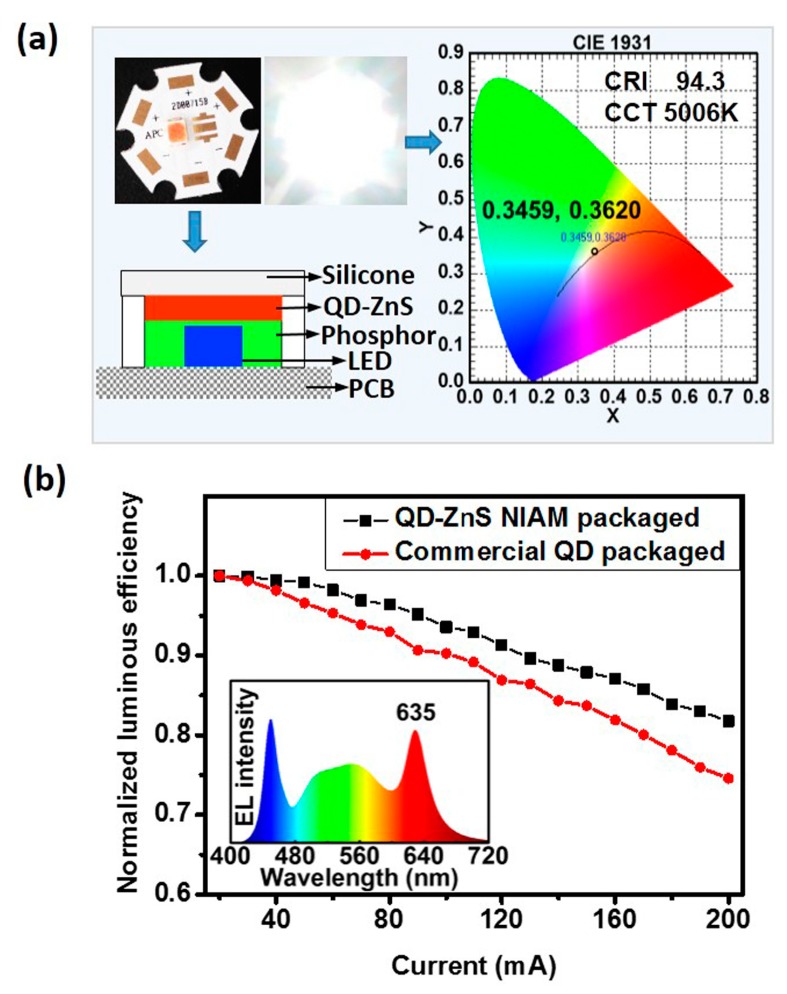
(**a**) The packaging structure of the as-prepared white-LED (WLED) with QD-ZnS NIAM and color information of the WLED prototype; (**b**) Luminous efficiency drop of the QD-ZnS NIAM and the common QD-packaged white-LEDs at different driving currents from 20 mA to 200 mA. (Inserted image shows the EL spectrum of the as-prepared white-LED with QD-ZnS NIAM).

A white-LED (WLED) prototype has been developed by employing hybrid LuAG:Ce phosphor and as-prepared red QD-ZnS NIAM. Figure 6a illustrates the LED package structure and color information of the WLED prototype. The as-prepared WLED has a correlated color temperature (CCT) of 5006 K and a color-rendering index (CRI) of 94.3. The relationship between the luminous efficiency of the LEDs and input forward current in the range of 20 mA to 200 mA (two times overdrive) and 3 V constant voltage is shown in Figure 6b. The as-prepared WLED with QD-ZnS NIAM presents a less than 20% efficiency drop, much better than the reference LED at the same CCT and CRI that is developed by using the QD thin film as color converter to replace QD-ZnS NIAM. The inserted image shows the electroluminescence (EL) spectra of the WLED prototype with QD-ZnS NIAM.

## 4. Conclusions

In summary, a QD-ZnS NIAM with reduced thermal fluorescent quenching and enhanced photochemical stability has been developed via a simple and reproducible approach. The NIAM structure reveals over 90% luminance maintained at 85 °C for QDs. Moreover, the QD-ZnS NIAM has been utilized as color converters, enabling on-chip packaging of QDs in LEDs. As-developed LED devices achieved enhanced PL stability and reduced thermal fluorescent quenching in comparison with the QD thin film. The QD-ZnS NIAM and the preparation method offer opportunities for economical fabrication of high-performance LEDs and other potential optoelectronic devices.

## Supplementary Materials

The following figures in Supplementary Materials are available online at www.mdpi.com/1996-1944/10/11/1242/s1, Figure S1: The XRD characterization of the ZnS nanosheet; Figure S2: The EDX measurement of the QD-ZnS NIAM.

## References

[1] Dasog M., Kehrle J., Rieger B., Veinot J.G.C. (2016). Silicon Nanocrystals and Silicon-Polymer Hybrids: Synthesis, Surface Engineering, and Applications. Angew. Chem. Int. Ed..

[2] Kagan C.R., Lifshitz E., Sargent E.H., Talapin D.V. (2016). Building devices from colloidal quantum dots. Science.

[3] Dai X.L., Zhang Z.X., Jin Y.Z., Niu Y., Cao H.J., Liang X.Y., Chen L.W., Wang J.P., Peng X.G. (2014). Solution-processed, high-performance light-emitting diodes based on quantum dots. Nature.

[4] Nan W.N., Niu Y.A., Qin H.Y., Cui F., Yang Y., Lai R.C., Lin W.Z., Peng X.G. (2012). Crystal Structure Control of Zinc-Blende CdSe/CdS Core/Shell Nanocrystals: Synthesis and Structure-Dependent Optical Properties. J. Am. Chem. Soc..

[5] Ou C., Jing Z., Chauhan V.P., Jian C., Cliff W., Harris D.K., He W., Hee-Sun H., Dai F., Jain R.K. (2013). Compact high-quality CdSe-CdS core-shell nanocrystals with narrow emission linewidths and suppressed blinking. Nat. Mater..

[6] Reiss P., Protiere M., Li L. (2009). Core/shell semiconductor nanocrystals. Small.

[7] Tomczak N., Janczewski D., Han M.Y., Vancso G.J. (2009). Designer polymer-quantum dot architectures. Prog. Polym. Sci..

[8] Kim S., Kim T., Kang M., Kwak S.K., Yoo T.W., Park L.S., Yang I., Hwang S., Lee J.E., Kim S.K. (2012). Highly Luminescent InP/GaP/ZnS Nanocrystals and Their Application to White Light-Emitting Diodes. J. Am. Chem. Soc..

[9] Li Z., Yao W., Kong L., Zhao Y., Li L. (2015). General Method for the Synthesis of Ultrastable Core/Shell Quantum Dots by Aluminum Doping. J. Am. Chem. Soc..

[10] Wang H.C., Lin S.Y., Tang A.C., Singh B.P., Tong H.C., Chen C.Y., Lee Y.C., Tsai T.L., Liu R.S. (2016). Mesoporous Silica Particles Integrated with All-Inorganic CsPbBr_3_ Perovskite Quantum-Dot Nanocomposites (MP-PQDs) with High Stability and Wide Color Gamut Used for Backlight Display. Angew. Chem. Int. Ed..

[11] Sun C., Zhang Y., Ruan C., Yin C.Y., Wang X.Y., Wang Y.D., Yu W.W. (2016). Efficient and Stable White LEDs with Silica-Coated Inorganic Perovskite Quantum Dots. Adv. Mater..

[12] Li Z., Kong L., Huang S., Li L. (2017). Highly Luminescent and Ultrastable CsPbBr_3_ Perovskite Quantum Dots Incorporated into a Silica/Alumina Monolith. Angew. Chem. Int. Ed..

[13] Peng X., Manna L., Yang W., Wickham J., Scher E., Kadavanich A., Alivisatos A.P. (2000). Shape control of CdSe nanocrystals. Nature.

[14] Kim F., Kwan S., Akana J., Yang P. (2001). Langmuir−Blodgett Nanorod Assembly. J. Am. Chem. Soc..

[15] Hu J., Odom T.W., Lieber C.M. (1999). Chemistry and Physics in One Dimension: Synthesis and Properties of Nanowires and Nanotubes. Acc. Chem. Res..

[16] Li J.P., Xu Y., Wu D., Sun Y.H. (2004). Hydrothermal synthesis of novel sandwich-like structured ZnS/octylamine hybrid nanosheets. Solid State Commun..

[17] Zhao Y.M., Riemersma C., Pietra F., Koole R., Donega C.D., Meijerink A. (2012). High-Temperature Luminescence Quenching of Colloidal Quantum Dots. ACS Nano.

[18] Cai X.C., Martin J.E., Shea-Rohwer L.E., Gong K., Kelley D.F. (2013). Thermal Quenching Mechanisms in II-VI Semiconductor Nanocrystals. J. Phys. Chem. C.

[19] Algar W.R., Kim H., Medintz I.L., Hildebrandt N. (2014). Emerging non-traditional Forster resonance energy transfer configurations with semiconductor quantum dots: Investigations and applications. Coord. Chem. Rev..

[20] Azevedo G., Monte A.F.G., Reis A.F., Messias D.N. (2014). Fluorescence resonance energy transfer measured by spatial photon migration in CdSe-ZnS quantum dots colloidal systems as a function of concentration. Appl. Phys. Lett..

[21] Zhou B., Xiao G.J., Yang X.Y., Li Q.J., Wang K., Wang Y.N. (2015). Pressure-dependent optical behaviors of colloidal CdSe nanoplatelets. Nanoscale.

[22] Tetsuka H., Ebina T., Mizukami F. (2008). Highly luminescent flexible quantum dot-clay films. Adv. Mater..

[23] Kim K., Woo J.Y., Jeong S., Han C.-S. (2011). Photoenhancement of a Quantum Dot Nanocomposite via UV Annealing and its Application to White LEDs. Adv. Mater..

