# Supplementary material for: Synthesis of Quantum Dot-ZnS Nanosheet Inorganic Assembly with Low Thermal Fluorescent Quenching for LED Application
Source: Materials (Basel). 2017 Oct 27;10(11):1242. doi: 10.3390/ma10111242 (PMC5706189; doi:10.3390/ma10111242)
Supplement: Supplementary file 1 [file materials-10-01242-s001.pdf]

Supplementary Materials: Synthesis of Quantum Dot-ZnS Nanosheet Inorganic Assembly with Low Thermal Fluorescent Quenching for LED Application

Yangyang Xie, Chong Geng, Yiqun Gao, Jay Guoxu Liu, Zi-Hui Zhang, Yonghui Zhang, Shu Xu and Wengang Bi

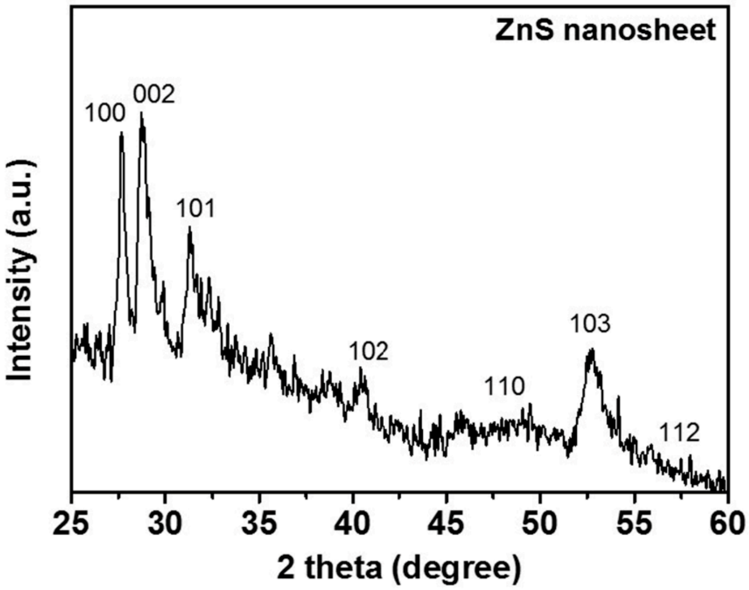

Figure S1. The XRD characterization of the ZnS nanosheet.

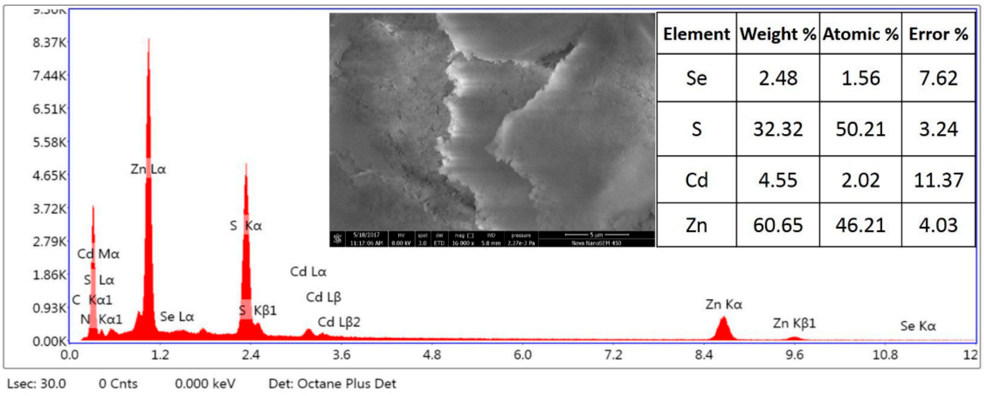

Element	Weight %	Atomic %	Error %
Se	2.48	1.56	7.62
S	32.32	50.21	3.24
Cd	4.55	2.02	11.37
Zn	60.65	46.21	4.03

Figure S2. The EDX measurement of the QD-ZnS NIAM (inserted images show the SEM pattern of the QDs-ZnS NIAM sample and the test data).

© 2017 by the authors. Submitted for possible open access publication under the terms and conditions of the Creative Commons Attribution (CC BY) license (http://creativecommons.org/licenses/by/4.0/).
